# TiNbSn stems with gradient changes of Young’s modulus and stiffness reduce stress shielding compared to the standard fit-and-fill stems

**DOI:** 10.1186/s40001-023-01199-z

**Published:** 2023-07-03

**Authors:** Kazuyoshi Baba, Yu Mori, Daisuke Chiba, Yoshiyuki Kuwahara, Hiroaki Kurishima, Hidetatsu Tanaka, Atsushi Kogure, Masayuki Kamimura, Norikazu Yamada, Susumu Ohtsu, Masamizu Oyama, Naoya Masahashi, Shuji Hanada, Eiji Itoi, Toshimi Aizawa

**Affiliations:** 1 Department of Orthopaedic Surgery, Tohoku University School of Medicine, 1-1 Seiryo-Machi, Aoba-ku, Sendai, Miyagi 980-8574 Japan; 2 Department of Orthopaedic Surgery, Sendai Red Cross Hospital, 2‑43‑3 Yagiyamahoncho, Taihaku‑ku, Sendai, Miyagi 982-8501 Japan; 3 Department of Orthopaedic Surgery, Osaki Citizen Hospital, 3-8-1 Furukawahonami, Osaki, Miyagi 989-6183 Japan; 4 Institute for Materials Research, Tohoku University, 2-1-1 Katahira, Aoba-ku, Sendai, Miyagi 980-8577 Japan; 5 Department of Orthopaedic Surgery, Tohoku Rosai Hospital, 4-3-21 Dainohara, Aoba-ku, Sendai, Miyagi 981-0911 Japan

**Keywords:** TiNbSn alloy, Total hip arthroplasty, Stress shielding, Low Young’s modulus, Functionally graded characteristics

## Abstract

**Background:**

The difference between Young’s moduli of the femur and the stem causes stress shielding (SS). TiNbSn (TNS) stem has a low Young’s modulus and strength with gradient functional properties during the change in elastic modulus with heat treatment. The aim of this study was to investigate the inhibitory effect of TNS stems on SS and their clinical outcomes compared to conventional stems.

**Methods:**

This study was a clinical trial. Primary THA was performed using a TNS stem from April 2016 to September 2017 for patients in the TNS group. Unilateral THA was performed using a Ti6Al4V alloy stem from January 2007 to February 2011 for patients in the control group. The TNS and Ti6Al4V stems were matched in shape. Radiographs were obtained at the 1- and 3-year follow-ups. Two surgeons independently checked the SS grade and appearance of cortical hypertrophy (CH). The Japanese Orthopaedic Association (JOA) scores before and 1 year after surgery were assessed as clinical scores.

**Results:**

None of the patients in the TNS group had grade 3 or 4 SS. In contrast, in the control group, 24% and 40% of patients had grade 3 and 4 SS at the 1- and 3-year follow-ups, respectively. The SS grade was lower in the TNS group than in the control group at the 1- and 3-year follow-ups (*p* < 0.001). The frequencies of CH in both groups were no significant difference at the 1- and 3-year follow-ups. The JOA scores of the TNS group significantly improved at 1 year after surgery and were comparable to control group.

**Conclusion:**

The TNS stem reduced SS at 1 and 3 years after THA compared to the proximal-engaging cementless stem, although the shapes of the stems matched. The TNS stem could reduce SS, stem loosening, and periprosthetic fractures.

*Trial registration*: Current Controlled Trials. ISRCTN21241251. https://www.isrctn.com/search?q=21241251. The date of registration was October 26, 2021. Retrospectively registered.

## Background

Total hip arthroplasty (THA) is a reliable surgical treatment for hip deformity and pain that improves hip pain and quality of life. In recent years, the number of patients undergoing THA has increased worldwide, with the number of patients under 65 years of age predicted to increase in the future because young and active patients who had hip degeneration too extensive for osteotomy demanded to regain full activities [1, 2]. Although THA has shown favourable clinical results, it can cause complications, such as aseptic loosening. Aseptic loosening of the femoral stem is the most common cause of THA revision [3, 4]. Young age at surgery is associated with a high revision risk. The lifetime revision risk is significantly higher in men aged 50–54 years than in those aged 70–74 years (29.6% vs. 7.7%) [5]. Preventing aseptic loosening is critical to improving the long-term outcome of THA.

Stress shielding (SS) affected aseptic loosening when combined with other risk factors such as polyethylene wear and low-precision installation of the prosthesis [3, 6]. Highly cross-linked polyethylene was developed to reduce polyethylene wear [7]. While improvements have been obtained for polyethylene wear, stress shielding of cementless hip prostheses is still an issue that needs to be improved.

The change of local strain caused by the difference in Young's moduli of bone and implant materials leads to SS [8]. Abnormal load transmission from the proximal portion of the stem to the area in contact with the stem tip causes cortical hypertrophy (CH) [9]. Excessive stiffness of the stem was one of the causes of SS, so an improved femoral stem material that combined low elastic modulus with strength was needed. The Young’s modulus of bone is 10–30 GPa, and the ideal Young's modulus of an implant material is similar to that of bone while maintaining sufficient strength [10]. Femoral stems made of materials with lower Young's moduli were previously developed to approximate Young’s modulus of bone [11]; however, their results were unacceptable because of poor strength. The femoral stem material should have biocompatibility and strength to fix the femur. Titanium alloys, particularly Ti6Al4V alloys, are most commonly used in the medical field and have adequate biocompatibility and corrosion resistance for use as orthopaedic implants [12]. In contrast, Young’s modulus of the Ti6Al4V alloy is approximately 110 GPa, while the stiffnesses of bone and the Ti6Al4V alloy differ [13, 14].

Hanada et al. developed the novel Ti-33.6Nb-4Sn (TNS) alloy, which has a low Young's modulus, the same tensile strength as Ti6Al4V, and functionally graded characteristics of an adjustable Young's modulus with heat treatment [15, 16]. A novel cementless femoral stem of the TNS alloy with Young's modulus gradient properties with heat treatment has been developed [16]. Furthermore, short-term clinical outcomes of TNS stems include a reduced incidence of SS [17].

However, the reduction of SS by TNS stems compared to similarly designed Ti6Al4V stems has not been investigated. The aim of this study was to evaluate the reduced incidence of SS and the postoperative clinical score of TNS stems compared to similarly designed Ti6Al4V stems.

## Materials and methods

### Patients

This was a multicentre, open-label, single-arm clinical trial. The Clinical Research Ethics Committee of Tohoku University Hospital approved the study protocol (approval no.: #201,506–1). The current clinical trial ID is ISRCTN21241251. Informed consent was obtained from all patients undergoing THA from April 2016 to September 2017. Patients awaiting unilateral THA were included in this trial and enrolled in the TNS group. Inclusion criteria were age over 20 years and a diagnosis of osteoarthritis, avascular necrosis, or rheumatoid arthritis. Exclusion criteria were the previous operation of the affected side of the hip (THA, osteotomy, or tenotomy around the hip joint), bilateral hip disorder, rheumatoid arthritis of Charnley category C (multiple joint diseases or other diseases limiting mobility) [18], a history of deep venous thrombosis or pulmonary embolism, metal allergy, severe obesity (body mass index > 35.0 kg/m^2^), uncontrolled diabetes mellitus, and infection around the hip joint. For the control group, due to the scarcity of patients undergoing THA with a similarly designed Ti6Al4V stem and the same surgical approach, we retrospectively reviewed the medical charts of a consecutive case series of patients who underwent unilateral THA with Ti6Al4V stem from January 2007 to February 2011. The inclusion criterion was a diagnosis of hip osteoarthritis or idiopathic osteonecrosis of the femoral head, and exclusion criteria were unavailable data in medical records or poor-quality radiographs.

### Characteristics of the TNS stem

**Fig. 1:**
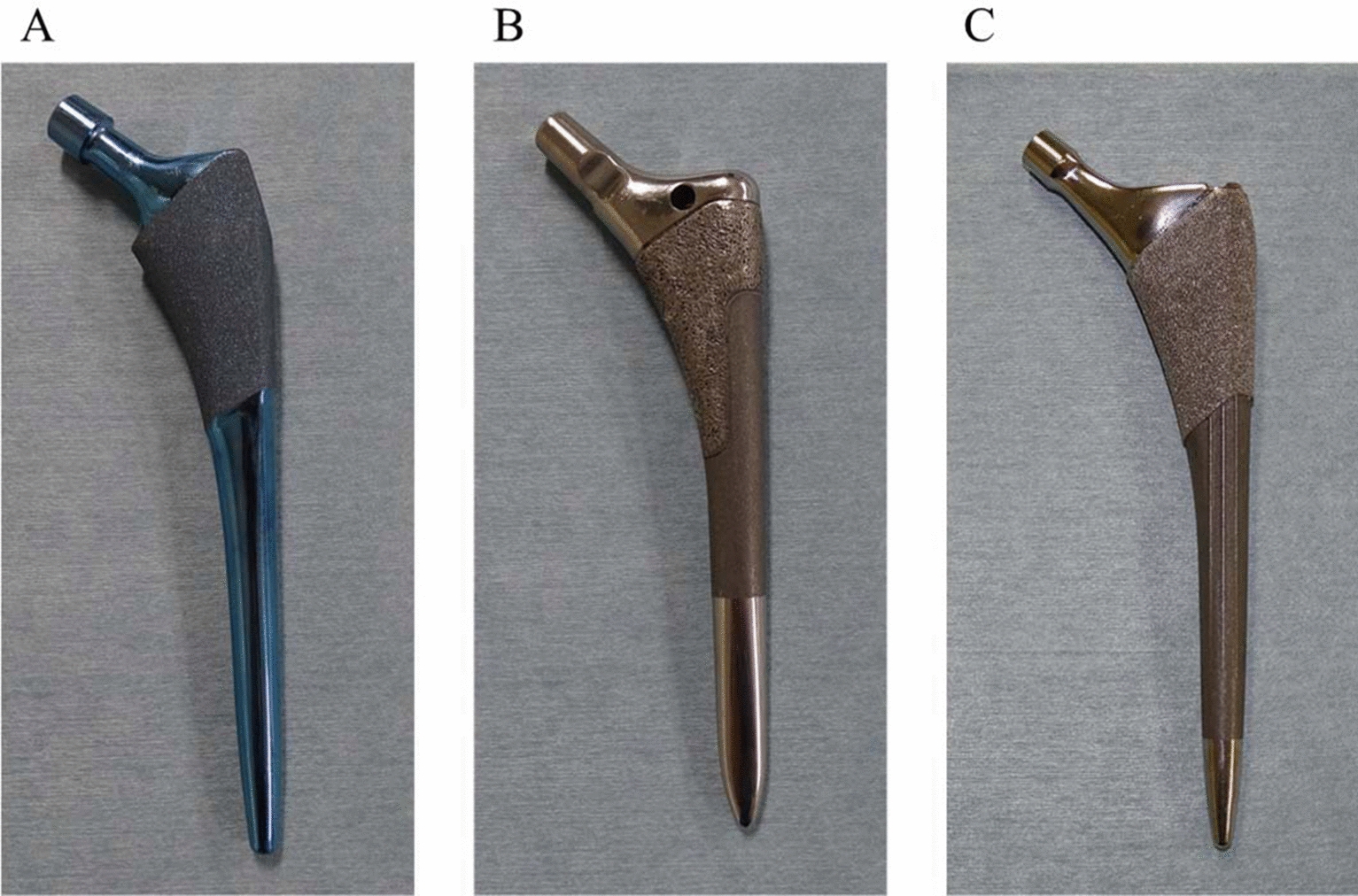
TiNbSn stems implanted in the control group. **A**. Overview of the TiNbSn stem. **B**. Overview of the VerSys HA/TCP Fiber Metal Taper stem. **C**. Overview of the Synergy Select II stem

Figure 1A shows the design of the TNS stem, categorized as a metaphyseal-filling stem [19]. The proximal one-third of the stem was processed to a rough surface with sandblasting, while the distal two-thirds was polished (Fig. 1A). The TNS stems were fabricated as reported previously [16, 17, 20, 21]. They were manufactured and provided by Mizuho Co., Tokyo, Japan. The cost of the clinical trial was covered by Mizuho Co., Tokyo, Japan.

### Surgery and rehabilitation

All patients underwent THA with the conventional posterolateral approach [22]. Six orthopaedic surgeons performed the surgeries at three institutions. The femur was scraped using a hand-powered reamer and broach. The TNS stem was inserted with the press fit technique. An ARC HA cup (Mizuho Co., Tokyo, Japan) was implanted in patients using the TNS stem. The stem used in the control group was VerSys HA/TCP Fiber Metal Taper (Zimmer, USA) or Synergy Select II (Smith & Nephew, England; Fig. 1B, C). The design of these stems was similar to that of the TNS stem and was classified as the metaphyseal-filling designs similar to TNS stems [19]. The control stems were mainly made of Ti6Al4V. The Trilogy (Zimmer, USA) or Reflection (Smith & Nephew, England) cup was implanted in the control group. From postoperative day 1, the patients were allowed to walk with full weight bearing.

### Radiographic evaluation

**Fig. 2:**
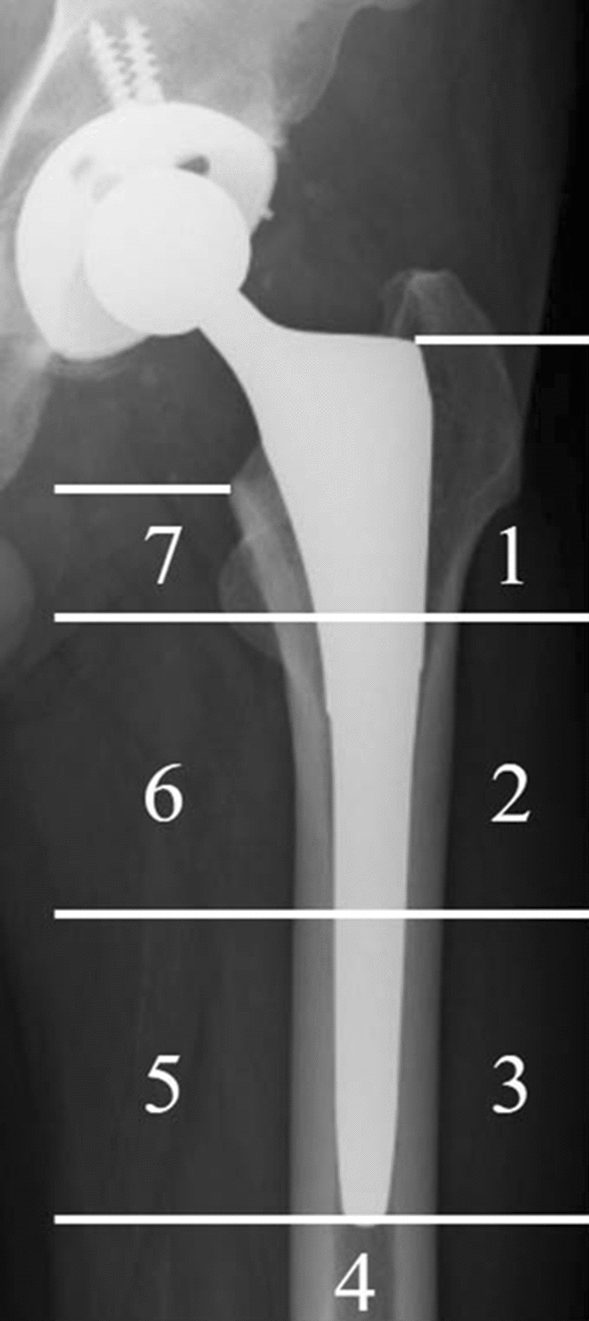
The assessment areas of stress shielding according to Gruen zone. The numbers indicate the Gruen zone

Anteroposterior radiographs of the bilateral hips and lateral radiographs of the affected hip were acquired preoperatively and immediately and 1 and 3 years postoperatively in both TNS and control groups. The incidence of SS was assessed with radiographs at 1 and 3 years postoperatively using Engh’s classification [23]. The frequency of SS at 3-year follow-up were also assessed according to Gruen zone 1–7 (Fig. 2) [24]. The incidence of CH was also evaluated. CH was defined as fusiform enlargement of the cortical bone in the area around the stem tip. Two orthopaedic surgeons who did not implant the stems performed radiologic assessments of SS and CH independently under blind conditions.

### Clinical assessment

The Japanese Orthopaedic Association (JOA) scores were used to assess the clinical outcomes preoperatively and 1 year postoperatively in both groups. The JOA hip scoring system is a 100-point scale that comprises subcategories of pain (40 points), range of motion (20 points), ability to walk (20 points), and activities of daily living (20 points) [25].

### Statistical analysis

All results are expressed as mean ± standard deviation. Patient characteristics of the TNS and control groups were analysed using the Wilcoxon signed-rank test. JOA scores were compared using the one-way analysis of variance with the Tukey–Kramer post hoc test (JMP 15, SAS Institute Japan Ltd., Tokyo, Japan). Kappa coefficients were calculated for the reproducibility of scores between examiners in the grades of the Engh's classification and CH using SPSS version 21 (IBM, Armonk, NY, USA) to assess the reliability of the measurements. Differences in the incidence of SS and CH and the frequency of SS according to Gruen zone were determined using Pearson's Chi-squared test. Statistical significance was set at *p* < 0.05.

## Results

### Patient characteristics

We enrolled 36 women and four men in the TNS group and 22 women and three men in the control group. The mean ages at operation in the TNS and control groups were 64.2 ± 10.7 and 64.1 ± 10.6 years, respectively (Table 1). A combination of the VerSys HA and TCP Fiber Metal Taper and Trilogy cup was used in 13 patients and that of Synergy Select II and Reflection cup was used in 12 patients. The preoperative diagnosis was osteoarthritis in 35 patients and idiopathic osteonecrosis of the femoral head in five patients in the TNS group and hip osteoarthritis in 21 patients and idiopathic osteonecrosis of the femoral head in four patients in the control group. Patient characteristics, including sex, age at surgery, body mass index, or pathogenic disease, did not differ between the TNS and control groups (Table 1).

**Table 1:** Demographics of patients

Variable	TNS group	Control group	*p* value
Number of patients (hips)	40	25	
Gender female:male, *n* (%)	36 (90): 4 (10)	22 (88): 3 (12)	*p* = 0.80
Mean age at surgery (years)	64.2 ± 10.7	64.1 ± 10.6	*p* = 0.91
Body mass index (kg/m^2^)	24.6 ± 4.1	24.3 ± 2.6	*p* = 0.89
Implant			
Stem, *n*	TNS stem: 40	Versys Taper: 13	
		Synergy select II: 12	
Cup, *n*	ARC HA Cup:40	Trilogy: 13	
		REFLECTION: 12	
Preoperative diagnosis, *n* (%)			
Osteoarthritis	35 (87.5)	21 (84)	*p* = 0.69
Idiopathic osteonecrosis of the femoral head	5 (12.5)	4 (16)	

Results are the mean with a standard deviation. One patient in TNS group was lost at 3-year follow-up

### Radiographic findings

Table 2 shows the comparison of the incidence and grade of SS between the two groups.

**Table 2:** Stress shielding according to Engh’s classification

Engh’s classification	TNS group		Control group	
	1-year follow-up Hips, (ratio)	3-year follow-up Hips, (ratio)	1-year follow-up Hips, (ratio)	3-year follow-up Hips, (ratio)
None	19, (47.5%)	14, (35%)	0, (0%)	0, (0%)
Grade 1	17, (42.5%)	10, (25%)	7, (28%)	5, (20%)
Grade 2	4, (10%)	16, (40%)	12, (48%)	10, (40%)
Grade 3	0, (0%)	0, (0%)	5, (20%)	9, (36%)
Grade 4	0, (0%)	0, (0%)	1, (4%)	1, (4%)

**Fig. 3:**
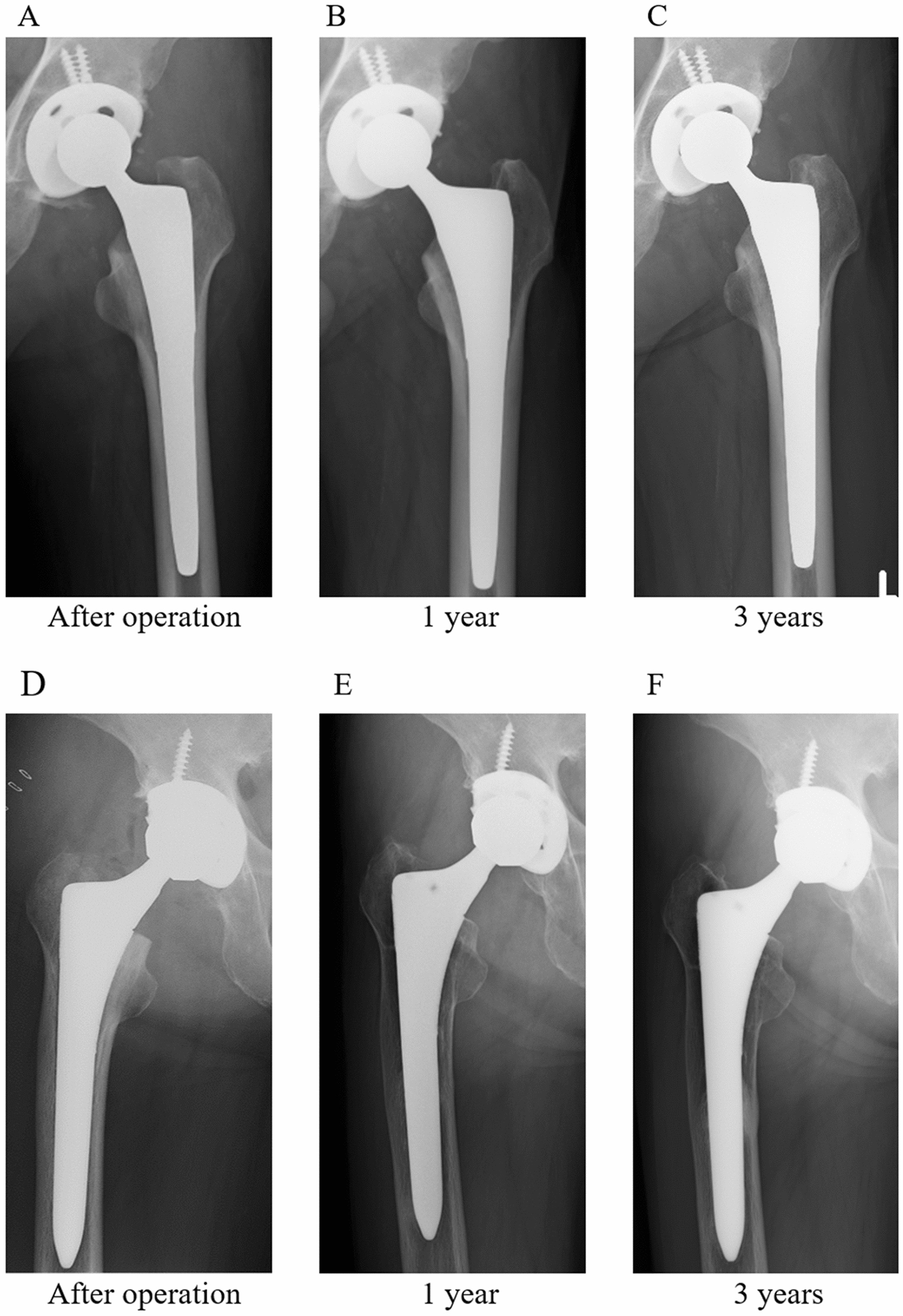
The reduction of stress shielding in the TiNbSn group and incidence of stress shielding in the control group. **A–C**. No apparent stress shielding was presented in the TiNbSn group immediately or 1 or 3 years postoperatively. **D–F**. Stress shielding was presented in the control group 1 year postoperatively but not immediately or 3 years postoperatively

Figure 3 shows changes in SS over time in both groups. The incidence and grade of SS were significantly lower in the TNS group than in the control group at the 1- and 3-year follow-ups (*p* < 0.001; Table 2). The interobserver reliability of the SS assessment was calculated. Kappa coefficients were 0.85 and 0.75 at the 1- and 3-year follow-ups in the TNS group, respectively, and 0.93 and 0.95 at the 1- and 3-year follow-ups in the control group, respectively. The reliability was almost in perfect agreement in both groups [26]. CHs were 2.5% and 2.6% at the 1- and 3-year follow-ups in the TNS group, respectively, and 8% and 12% at the 1- and 3-year follow-ups in the control group, respectively (Table 3). The results of SS assessment according to Gruen zone are shown in Table 4. Zone 1 and 7 were the regions where SS was observed in TNS and Control groups. The frequency of SS was no difference between two groups in zone 1 and 7. However, in zone 2 and 6, SS was observed frequently in Control group (Table 4).

**Table 3:** Assessment of cortical hypertrophy

Cortical hypertrophy	TNS group		Control group	
	1-year follow-up Hips, (ratio)	3-year follow-up Hips, (ratio)	1-year follow-up Hips, (ratio)	3-year follow-up Hips, (ratio)
	1, (2.5%)	1, (2.6%)	2, (8%)	3, (12%)

**Table 4:** Stress shielding according to Gruen zone

Gruen zone	TNS group	Control group	*p*
Zone 1	9, (23%)	11, (44%)	0.07
Zone 2	0, (0%)	6, (24%)	0.001^*^
Zone 3	0, (0%)	2, (8%)	0.07
Zone 4	0, (0%)	0, (0%)	–
Zone 5	0, (0%)	1, (4%)	0.2
Zone 6	1, (2.5%)	9, (36%)	< 0.001^*^
Zone 7	21, (53.8%)	19, (76%)	0.07

^*^p < 0.05

The incidence of CH did not differ significantly between the TNS and control groups at the 1- or 3-year follow-up. The interobserver reliability of the CH assessment was calculated. Kappa coefficients were 1.0 at the 1- and 3-year follow-ups in both groups. The reliability of the scale was excellent.

### Clinical assessment

**Fig. 4:**
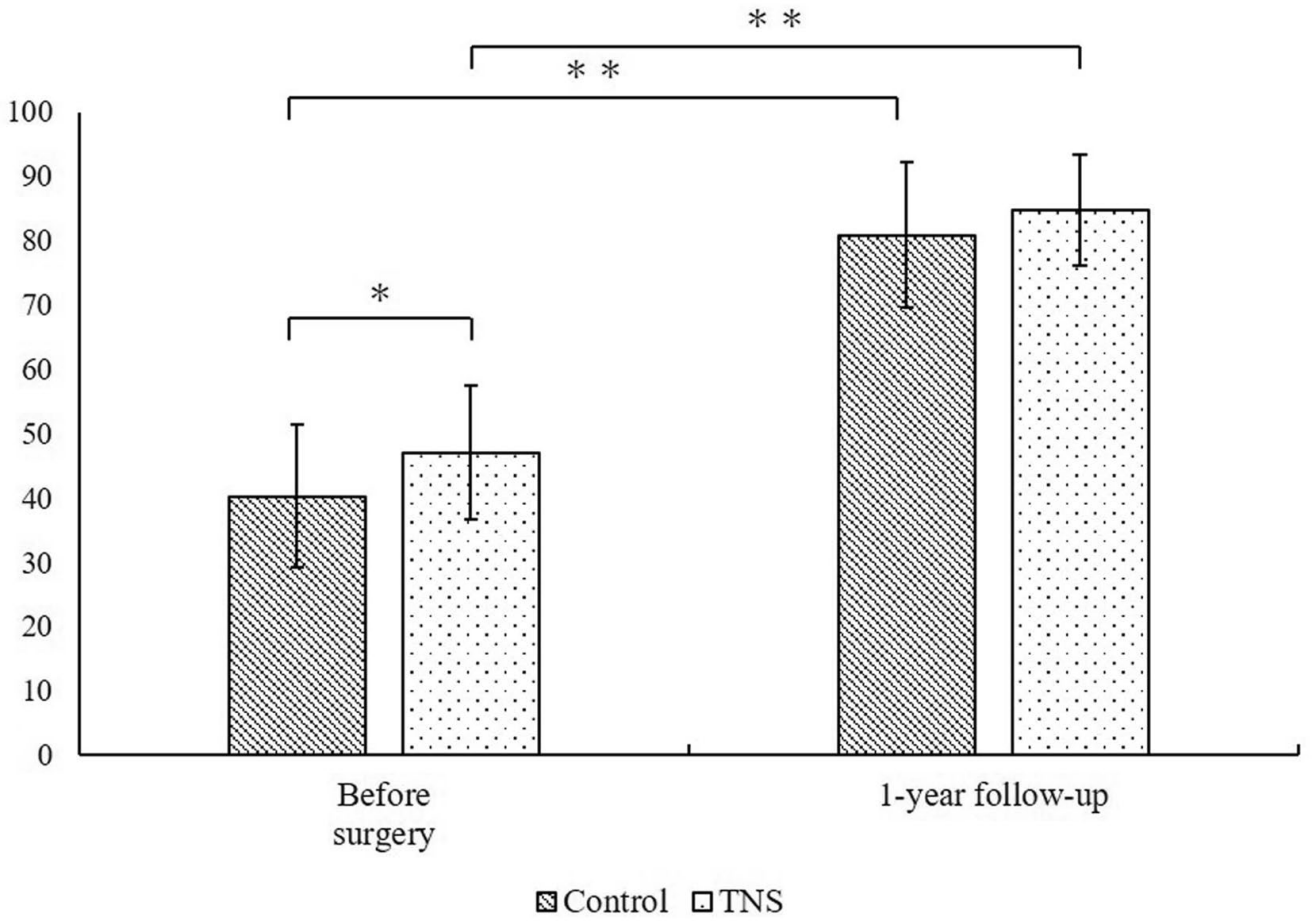
Comparison of the preoperative and 1-year postoperative Japanese Orthopaedic Association Hip score. *p < 0.05, Wilcoxon signed-rank test. **p < 0.0001, Wilcoxon signed-rank test

Preoperative JOA scores were 47.1 ± 10.4 and 40.2 ± 11.1 in the TNS and control groups, respectively, showing a statistically significant difference (Fig. 4). JOA scores at the 1-year follow-up were 84.7 ± 8.6 and 80.9 ± 11.4 in the TNS and control groups, respectively, showing no significant difference. JOA scores in the TNS and control groups improved after THA. No safety issues were observed.

## Discussion

This was the first study determining the severity of SS by comparing TNS and similarly designed Ti6Al4V stems. The TNS stem prevented SS in the short term. The clinical results of the TNS stem were acceptable compared to the metaphyseal-engaging cementless Ti6Al4V stems currently in use.

SS is a mechanical phenomenon that occurs after stem implantation into the femur and is caused by multiple factors, including stem stiffness, area of surface treatment, and large stem size compared to the femur size [27–29], with the key factor being the difference in elastic moduli of the stem and bone [30]. SS was not necessary associated with complications and clinical results [23, 31]. However, potential risks of SS were related with stem loosening, deficient bone stock when a revision surgery was required [32, 33]. The reduction of SS is critical to obtain the better clinical results of THA. Several animal models have suggested that a low elastic modulus of the stem leads to lesser resorption of the cortical area than a high elastic modulus of the stem [34, 35]. To resolve the mismatch in elastic moduli of the stem and bone, Robert Mathys developed an isoelastic stem as a low-elastic stem to avoid SS [36, 37]. However, the results with this stem were unacceptable [38], probably because of inadequate primary fixation. A femoral stem made of the TNS alloy, which has both low Young's modulus and comparable strength to the Ti6Al4V alloy, has been developed, and good short-term results have been reported [17]. In the present study, we compared TNS and conventional Ti6Al4V stems, and the radiological assessment indicated that TNS stems with a low Young's modulus had a superior effect on the suppression of SS compared to Ti6Al4V stems. The frequency of CH was higher in the TNS group at 3-year follow-up comparing at 1-year follow-up, however the actual number of cases of CH did not increase. This was because one patient in the TNS group dropped out of the study during the follow-up.

Femoral components of THA comprise articulation, structure, and fixation, with different mechanical properties. The requisite of the structural component, which couples the articulation and fixation components, is sufficient strength to overcome peak and dynamic stresses. When a stem is manufactured from a single alloy, such as the Ti6Al4V alloy, the alloy is selected to produce the stem according to the stiffness and Young’s modulus of the part that requires the most strength. The Ti6Al4V alloy is preferable as a biomedical material for stems because of its excellent biocompatibility and corrosion resistance. In contrast, its Young’s modulus is significantly higher than that of human cortical bone, leading to an imbalance in loading between the bone and the stem. The grade of SS caused by fit-and-fill stems implanted in the control group were reported previously using Engh’s classification. Kato reported that Synergy stems were shown grade 2 of SS appeared 66% and grade 3 and 4 of SS appeared 22% at 5-year follow-up [39]. Nishino also reported that Synergy stems had grade 2 of SS appeared 38% and grade 3 and 4 of SS appeared 46% at 10- to 12-year follow-up [40]. VerSys HA/TCP Fiber Metal Taper had 42% of them were shown grade 2 of SS, 7% were shown grade 3 of SS and degree 4 of SS was not appeared at 3-year follow-up [41]. Synergy stem and VerSys HA/TCP Fiber Metal Taper caused grade 2 and 3 of SS 3 to 10 years after THA. These results previously reported were consistent with our results of control stems. In contrast, the TNS stem exhibited novel properties and a gradient change in stiffness in the same alloy with heat treatment. Stiffness could be altered in the appropriate portion of the stem to utilize this property. The results of this study suggest that the TNS stem adequately transmitted the load to the proximal femur.

The low Young's modulus of the TNS alloy makes it useful as orthopaedic implants. Animal models have shown it to be a promising material for fracture treatment devices. Fracture healing is multifaceted, and besides material advantage, stem cell differentiation and proper inflammation are required for bone repair [42]. Mouse and rabbit tibial models have shown that TNS intramedullary nails are more effective than the Ti6Al4V alloy or stainless-steel intramedullary nails in promoting bone healing [43–46]. The low Young's modulus of the TNS alloy promotes the expression of runt-related transcription factor 2, which is the key signal for osteoblast differentiation at the fracture callus site and promotes bone healing [43]. Furthermore, the effect of the anodic oxidation method on the biocompatibility of the TNS alloy has been studied. The TNS alloy treated by anodic oxidation with sulfuric or acetic acid generates hydroxyapatite on its surface in vivo, which enhances bone conduction ability [47–52]. Osteoblast progenitor cells are expressed in the endosteum of the bone marrow [42]. The TNS alloy after anodic oxidation treatment promotes bone formation by proliferating and activating osteoblasts in the endosteum of the bone and exerts an antibacterial effect through photocatalytic performance owing to sodium tartrate and sulfuric acid [53–55]. The TNS alloy is considered to be a promising orthopaedic implant material owing to the low Young’s modulus and functional improvement by surface modification.

This study has several limitations. First, the control group did not undergo THA during the same period as the TNS group, and two types of stems were implanted in the control group. The methods or sites of stem surface treatments did not match completely with the TNS stem. Young's modulus of stems was not measured in the control group. It was ideal that Ti6Al4V stem having the completely same shape and surface treatment of the TNS stem was implanted as control. However, this was the first trial of TNS stem in clinical use. It was an ethical problem that the Ti6Al4V stems completely matched in shape and surface treatment were implanted in the control group. We aimed to compare the stems in current use having the concept of metaphyseal-filling stems focused on stem shape. The shape of the TNS stem was categorized as metaphyseal-filling or fit-and-fill stem. The number of control group did not completely match because the control group was selected retrospectively. Second, the SS was not evaluated quantitatively, such as evaluating bone mineral density of femurs. We did not evaluate bone mineral density of femurs. SS was evaluated using Engh’s classification and Gruen zone for comparison with previous studies using radiographic analyses of control stems implanted in this study [39–41]. Third, different surgeons performed the surgeries in the TNS and control groups at different timing. In both groups, surgeons with over 10 years of experience performed the surgery with the same approach; however, the surgical techniques were not completely same. In this report, TNS stems and control stems were not implanted by same surgeons and not in the same period. Finally, the sample size was small. THA with the TNS stem was performed as a clinical trial; therefore, only 40 patients could be followed up. Future studies with a large sample size are required. The follow-up period was short. Although SS was prevented in the short term, whether or not it could be prevented in the long term requires further investigation. We believe that safety and durability should be examined in the future.

## Conclusions

The TNS stem reduced SS at 1 and 3 years after THA compared to the proximal-engaging cementless stem, although the shape of the TNS stem was matched. The TNS stem could reduce SS, stem loosening, and periprosthetic fractures. Further studies are needed to determine whether a low Young’s modulus TNS stem can reduce stress shielding and improve long-term outcomes of THA.

## Data Availability

All data generated or analysed during this study are included in this published article.

## Abbreviations

THA: Total hip arthroplasty
SS: Stress shielding
CH: Cortical hypertrophy
TNS: Ti-33.6Nb-4Sn
JOA: Japanese Orthopaedic Association

## References

[1] Daras M, Macaulay W (2009). Total hip arthroplasty in young patients with osteoarthritis. Am J Orthop.

[2] Kurtz SM, Lau E, Ong K, Zhao K, Kelly M, Bozic KJ (2009). Future young patient demand for primary and revision joint replacement: national projections from 2010 to 2030. Clin Orthop Relat Res.

[3] Sundfeldt M, Carlsson LV, Johansson CB, Thomsen P, Gretzer C (2006). Aseptic loosening, not only a question of wear: a review of different theories. Acta Orthop.

[4] Ferguson RJ, Palmer AJ, Taylor A, Porter ML, Malchau H, Glyn-Jones S (2018). Hip replacement. Lancet.

[5] Bayliss LE, Culliford D, Monk AP, Glyn-Jones S, Prieto-Alhambra D, Judge A, Cooper C, Carr AJ, Arden NK, Beard DJ, Price AJ (2017). The effect of patient age at intervention on risk of implant revision after total replacement of the hip or knee: a population-based cohort study. Lancet.

[6] Yamako G, Chosa E, Zhao X, Totoribe K, Watanabe S, Sakamoto T, Nakane N (2014). Load-transfer analysis after insertion of cementless anatomical femoral stem using pre- and post-operative CT images based patient-specific finite element analysis. Med Eng Phys.

[7] Lewis G (2001). Properties of crosslinked ultra-high-molecular-weight polyethylene. Biomaterials.

[8] Huiskes R, Weinans H, Van Rietbergen B (1992). The relationship between stress shielding and bone resorption around total hip stems and the effects of flexible materials. Clin Orthop Relat Res.

[9] Lewis JL, Askew MJ, Wixson RL, Kramer GM, Tarr RR (1984). The influence of prosthetic stem stiffness and of a calcar collar on stresses in the proximal end of the femur with a cemented femoral component. J Bone Joint Surg Am.

[10] Apostu D, Lucaciu O, Berce C, Lucaciu D, Cosma D (2018). Current methods of preventing aseptic loosening and improving osseointegration of titanium implants in cementless total hip arthroplasty: a review. J Int Med Res.

[11] Bombelli R, Mathys R (1982). Cementless isoelastic RM total hip prosthesis. J R Soc Med.

[12] Mohammed MT, Khan ZA, Siddiquee AN (2014). Beta titanium alloys: the lowest elastic modulus for biomedical applications: a review. Int J Chem Mol Nucl Mater Metall Eng.

[13] Niinomi M, Nakai M (2011). Titanium-based biomaterials for preventing stress shielding between implant devices and bone. Int J Biomater.

[14] Niinomi M (1998). Mechanical properties of biomedical titanium alloys. Mater Sci Eng A.

[15] Miura K, Yamada N, Hanada S, Jung T-K, Itoi E (2011). The bone tissue compatibility of a new Ti–Nb–Sn alloy with a low Young’s modulus. Acta Biomater.

[16] Hanada S, Masahashi N, Jung T-K, Yamada N, Yamako G, Itoi E (2014). Fabrication of a high-performance hip prosthetic stem using β Ti–336 Nb–4Sn. J Mech Behav Biomed Mater.

[17] Chiba D, Yamada N, Mori Y, Oyama M, Ohtsu S, Kuwahara Y, Baba K, Tanaka H, Aizawa T, Hanada S, Itoi E (2021). Mid-term results of a new femoral prosthesis using Ti-Nb-Sn alloy with low Young’s modulus. BMC Musculoskelet Disord.

[18] Charnley J (1972). The long-term results of low-friction arthroplasty of the hip performed as a primary intervention. J Bone Joint Surg Br.

[19] Khanuja HS, Vakil JJ, Goddard MS, Mont MA (2011). Cementless femoral fixation in total hip arthroplasty. J Bone Joint Surg Am.

[20] Yamako G, Janssen D, Hanada S, Anijs T, Ochiai K, Totoribe K, Chosa E, Verdonschot N (2017). Improving stress shielding following total hip arthroplasty by using a femoral stem made of β type Ti-33.6 Nb-4Sn with a Young’s modulus gradation. J Biomech.

[21] Yamako G, Chosa E, Totoribe K, Hanada S, Masahashi N, Yamada N, Itoi E (2014). In-vitro biomechanical evaluation of stress shielding and initial stability of a low-modulus hip stem made of β type Ti-33.6 Nb-4Sn alloy. Med Eng Phys.

[22] Moore AT (1957). The self-locking metal hip prosthesis. J Bone Joint Surg Am.

[23] Engh CA, Bobyn JD, Glassman AH (1987). Porous-coated hip replacement: the factors governing bone ingrowth, stress shielding, and clinical results. J Bone Joint Surg Br.

[24] Gruen TA, McNeice GM, Amstutz HC (1979). “Modes of failure” of cemented stem-type femoral components: a radiographic analysis of loosening. Clin Orthop Relat Res.

[25] Imura S (1995). The Japanese Orthopaedic Association: evaluation chart of hip joint functions. J Jpn Orthop Assoc.

[26] Landis JR, Koch GG (1977). The measurement of observer agreement for categorical data. Biometrics.

[27] Bobyn JD, Mortimer ES, Glassman AH, Engh CA, Miller JE, Brooks CE (1992). Producing and avoiding stress shielding: laboratory and clinical observations of noncemented total hip arthroplasty. Clin Orthop Relat Res.

[28] Engh CA, Bobyn JD (1988). The influence of stem size and extent of porous coating on femoral bone resorption after primary cementless hip arthroplasty. Clin Orthop Relat Res.

[29] Kärrholm J, Anderberg C, Snorrason F, Thanner J, Langeland N, Malchau H, Herberts P (2002). Evaluation of a femoral stem with reduced stiffness: a randomized study with use of radiostereometry and bone densitometry. J Bone Joint Surg Am.

[30] Sumner DR (2015). Long-term implant fixation and stress-shielding in total hip replacement. J Biomech.

[31] Engh CA, Young AM, Engh CA, Hopper RH (2003). Clinical consequences of stress shielding after porous-coated total hip arthroplasty. Clin Orthop Relat Res.

[32] Savio D, Bagno A (2022). When the total hip replacement fails: a review on the stress-shielding effect. Processes.

[33] Sanli I, Arts JJ, Geurts J (2016). Clinical and radiologic outcomes of a fully hydroxyapatite-coated femoral revision stem: excessive stress shielding incidence and its consequences. J Arthroplasty.

[34] Turner TM, Sumner DR, Urban RM, Igloria R, Galante JO (1997). Maintenance of proximal cortical bone with use of a less stiff femoral component in hemiarthroplasty of the hip without cement: an investigation in a canine model at six months and two years. J Bone Joint Surg Am.

[35] Bobyn JD, Glassman AH, Goto H, Krygier JJ, Miller JE, Brooks CE (1990). The effect of stem stiffness on femoral bone resorption after canine porous-coated total hip arthroplasty. Clin Orthop Relat Res.

[36] Morscher E, Mathys R (1975). First experiences with a cementless isoelastic total hip prosthesis. Z Orthop Ihre Grenzgeb.

[37] Morscher E, Mathys R (1974). Total isoelastic hip prosthesis implanted without cement: initial results. Acta Orthop Belg.

[38] Trebse R, Milosev I, Kovac S, Mikek M, Pisot V (2005). Poor results from the isoelastic total hip replacement: 14–17-year follow-up of 149 cementless prostheses. Acta Orthop.

[39] Kato S, Nozawa M, Kim S, Sakamoto Y, Ochi H, Ishijima M (2022). Comparison of the 5-year outcomes between standard and short fit-and-fill stems in Japanese populations. Arthroplasty Today.

[40] Nishino T, Mishima H, Kawamura H, Shimizu Y, Miyakawa S, Ochiai N (2013). Follow-up results of 10–12 years after total hip arthroplasty using cementless tapered stem—frequency of severe stress shielding with synergy stem in Japanese patients. J Arthroplasty.

[41] Inaba Y, Kobayashi N, Oba M, Ike H, Kubota S, Saito T (2016). Difference in postoperative periprosthetic bone mineral density changes between 3 major designs of uncemented stems: a 3-year follow-up study. J Arthroplasty.

[42] Mori Y, Adams D, Hagiwara Y, Yoshida R, Kamimura M, Itoi E, Rowe DW (2016). Identification of a progenitor cell population destined to form fracture fibrocartilage callus in Dickkopf-related protein 3-green fluorescent protein reporter mice. J Bone Miner Metab.

[43] Mori Y, Fujisawa H, Kamimura M, Kogure A, Tanaka H, Mori N, Masahashi N, Aizawa T (2021). Acceleration of fracture healing in mouse tibiae using intramedullary nails composed of β-Type TiNbSn alloy with low Young's modulus. Tohoku J Exp Med.

[44] Kogure A, Mori Y, Tanaka H, Kamimura M, Masahashi N, Hanada S, Itoi E (2019). Effects of elastic intramedullary nails composed of low Young's modulus Ti-Nb-Sn alloy on healing of tibial osteotomies in rabbits. J Biomed Mater Res B Appl Biomater.

[45] Ito K, Mori Y, Kamimura M, Koguchi M, Kurishima H, Koyama T, Mori N, Masahashi N, Hanada S, Itoi E, Aizawa T (2022). β-type TiNbSn alloy plates with low young modulus accelerates osteosynthesis in rabbit tibiae. Clin Orthop Relat Res.

[46] Fujisawa H, Mori Y, Kogure A, Tanaka H, Kamimura M, Masahashi N, Hanada S, Itoi E (2018). Effects of intramedullary nails composed of a new β-type Ti-Nb-Sn alloy with low Young's modulus on fracture healing in mouse tibiae. J Biomed Mater Res B Appl Biomater.

[47] Tanaka H, Mori Y, Noro A, Kogure A, Kamimura M, Yamada N, Hanada S, Masahashi N, Itoi E (2016). apatite formation and biocompatibility of a low Young's modulus Ti-Nb-Sn alloy treated with anodic oxidation and hot water. PLoS ONE.

[48] Mori Y, Mori N, Aizawa T (2022). Improving osteoinductive properties and imparting antibacterial activity to titanium alloys. J Bone Miner Metab.

[49] Mori Y, Masahashi N, Aizawa T (2022). A review of anodized TiNbSn alloys for improvement in layer quality and application to orthopedic implants. Materials.

[50] Masahashi N, Mori Y, Tanaka H, Kogure A, Inoue H, Ohmura K, Kodama Y, Nishijima M, Itoi E, Hanada S (2019). Bioactive TiNbSn alloy prepared by anodization in sulfuric acid electrolytes. Mater Sci Eng C Mater Biol Appl.

[51] Masahashi N, Mori Y, Tanaka H, Kogure A, Inoue H, Ohmura K, Kodama Y, Nishijima M, Itoi E, Hanada S (2017). Study of bioactivity on a TiNbSn alloy surface. Thin Solid Films.

[52] Kunii T, Mori Y, Tanaka H, Kogure A, Kamimura M, Mori N, Hanada S, Masahashi N, Itoi E (2019). Improved osseointegration of a TiNbSn alloy with a low Young’s modulus treated with anodic oxidation. Sci Rep.

[53] Masahashi N, Mori Y, Kurishima H, Inoue H, Mokudai T, Semboshi S, Hatakeyama M, Itoi E, Hanada S (2021). Photoactivity of an anodized biocompatible TiNbSn alloy prepared in sodium tartrate/hydrogen peroxide aqueous solution. Appl Surf Sci.

[54] Kurishima H, Mori Y, Ishii K, Inoue H, Mokudai T, Fujimori S, Itoi E, Hanada S, Masahashi N, Aizawa T (2022). Antibacterial activity of an anodized TiNbSn alloy prepared in sodium tartrate electrolyte. Front Bioeng Biotechnol.

[55] Mori Y, Fujimori S, Kurishima H, Inoue H, Ishii K, Kubota M, Kawakami K, Mori N, Aizawa T, Masahashi N (2023). Antimicrobial properties of TiNbSn alloys anodized in a sulfuric acid electrolyte. Materials.

